# Antibiofilm Effects of Plant Extracts Against *Staphylococcus aureus*

**DOI:** 10.3390/microorganisms13020454

**Published:** 2025-02-19

**Authors:** Alexia Barbarossa, Antonio Rosato, Roberta Tardugno, Antonio Carrieri, Filomena Corbo, Francesco Limongelli, Luciana Fumarola, Giuseppe Fracchiolla, Alessia Carocci

**Affiliations:** 1 Department of Pharmacy-Pharmaceutical Sciences, University of Bari “Aldo Moro”, 70125 Bari, Italy; alexia.barbarossa@uniba.it (A.B.); roberta.tardugno@uniba.it (R.T.); antonio.carrieri@uniba.it (A.C.); filomena.corbo@uniba.it (F.C.); francesco.limongelli@uniba.it (F.L.); giuseppe.fracchiolla@uniba.it (G.F.); 2 Interdisciplinary Department of Medicine, School of Medicine, University of Bari “Aldo Moro”, 70124 Bari, Italy; luciana.fumarola@uniba.it

**Keywords:** plant extracts, antibacterial resistance, XTT method, bacterial biofilm, *Staphylococcus aureus* strains

## Abstract

The global rise in antimicrobial resistance poses a significant threat to public health, necessitating alternative therapeutic options. One critical challenge is treating infections caused by biofilm-forming bacteria, which are notably resistant to conventional antibiotics. *Staphylococcus aureus*, including methicillin-resistant strains (MRSA), is a major pathogen in biofilm-related infections, complicating treatment and leading to chronic cases. Plant extracts have emerged as promising alternatives, offering new avenues for effective treatment. This study evaluated the antibacterial and antibiofilm activities of commercial extracts of *Vitis vinifera* L. (grape), *Camellia sinensis* L. (green tea), *Olea europaea* L. (olive), *Quercus robur* (oak), and *Coffea arabica* L. (coffee) against *S. aureus* strains from ATCC collections and clinical isolates. Preliminary screening using the disk diffusion test assessed the zones of inhibition, which was followed by minimum inhibitory concentration (MIC) determination via broth microdilution, with *Quercus robur* L. showing the best overall MIC results. The results obtained demonstrate the strong antibacterial activity of the extracts, with the MIC values ranging from 0.2 to 12.4 mg/mL. Using the XTT reduction assay, the extracts inhibited biofilm growth by 80–85% after 24 h of incubation, with *Coffea arabica* L. achieving interesting antibiofilm activities. These findings suggest that the investigated plant extracts hold potential as antimicrobial agents and biofilm inhibitors, offering an alternative approach to tackling antimicrobial resistance. Further research is needed to explore their potential applications in developing novel adjuvant therapies.

## 1. Introduction

The dramatic rise in antimicrobial resistance (AMR) among pathogenic bacteria is a serious concern for human health [1]. It occurs through the antibiotic selective pressure mechanism. This mechanism destroys all bacteria, only allowing the survival of those species that have a mutation that makes them resistant to the action of the drug. The result is the emergence of a subpopulation of bacteria resistant to the action of the antibiotic, promoting super-infections that are more difficult to treat pharmacologically. The situation continues to worsen, despite efforts to curb antibiotic resistance. Indeed, AMR increases medical costs, hospital stays, and mortality. *Staphylococcus aureus*, a key pathogen of the microbial world, stands out as one of the most concerning multi-resistant pathogens confronting modern medicine. With its ability to rapidly develop resistance mechanisms against a wide array of antibiotics, *S. aureus* poses a significant challenge to healthcare systems worldwide [2]. This bacterium’s adaptability and resilience have fueled its prominence in hospital-acquired infections, community-associated diseases, and veterinary settings, further complicating treatment regimens and patient outcomes [3]. Of particular concern is its ability to form biofilms, intricate communities of bacteria encased in a self-produced matrix, which not only enhances its resistance to antibiotics but also promote chronic and recurrent infections [4]. Biofilm formation not only enhances the bacteria’s resistance to antibiotics but also causes infections that are particularly challenging to treat, often leading to chronic infections. This issue is further exacerbated by the rise of methicillin-resistant *S. aureus* (MRSA) strains, which are notoriously difficult to eradicate [5]. In the face of this formidable pathogen, exploring alternative therapeutic approaches becomes imperative to mitigating the escalating threat of multidrug resistance.

Currently, international research is reporting on non-traditional antimicrobials, such as plant extracts, and their potential use against life-threatening resistant bacterial infections [6]. In recent years, plant extracts have gained attention as potential alternatives in the fight against *S. aureus* infections, including those involving biofilms [7]. Plant-derived compounds are known for their diverse bioactive properties, including antibacterial, anti-inflammatory, and antioxidant activities. Unlike conventional antibiotics, which often target a specific bacterial process, plant extracts can exert multifaceted effects on bacterial cells, potentially reducing the risk of resistance development [8]. Additionally, the natural origin and low toxicity of plant extracts make them attractive candidates for developing new therapeutic strategies [9,10]. Research has indicated that various plant extracts possess bioactive compounds that can disrupt bacterial cell processes [11,12,13]. *Vitis vinifera* L., *Camellia sinensis* L., *Olea europaea* L., *Quercus robur* L., and *Coffea arabica* L. have been extensively studied for their antibacterial properties. Plant extracts have garnered attention for their potential antimicrobial properties, offering a promising avenue for addressing the limitations of conventional antibiotics [14,15]. These natural compounds not only exhibit antimicrobial activity but also possess diverse bioactive molecules capable of disrupting biofilm formation and enhancing immune responses. Certainly, the extracts derived from *V. vinifera* L. (grape seed), *C. sinensis* L. (green tea), *Olea europaea* L. (olive), *Q. robur* (oak), and *C. arabica* L. (coffee) have shown promising antimicrobial activity against *S. aureus* biofilms. Indeed, *V. vinifera* L. extract, commonly known as grapevine, obtained from grape seeds, is rich in polyphenolic compounds such as proanthocyanidins and resveratrol. These compounds possess potent antimicrobial properties and have been found to inhibit biofilm formation and reduce the virulence factors associated with *S. aureus*. Additionally, grape seed extract can disrupt preformed biofilms, rendering the bacteria more susceptible to antimicrobial agents [16]. *C. sinensis* L., commonly known as green tea, contains catechins, particularly epigallocatechin gallate (EGCG), which exhibit strong antibacterial activity against *S. aureus*. EGCG disrupts biofilm formation and inhibits the expression of genes involved in biofilm production, thereby reducing the virulence of *S. aureus* infections [17]. *O. europaea* L. extract, derived from olive leaves, is rich in phenolic compounds such as oleuropein and hydroxytyrosol. These compounds possess antibacterial properties and have been shown to inhibit biofilm formation by *S. aureus* [18]. Moreover, olive leaf extract can enhance the activity of conventional antibiotics against *S. aureus* biofilms, potentially overcoming antibiotic resistance [19]. *Quercus robur*, or oak extract, rich in tannins and polyphenolic compounds, disrupts *S. aureus* biofilm formation and disperses established biofilms, enhancing their susceptibility to antimicrobials [20]. *C. arabica* L. extract, containing chlorogenic acid and other phenolics, inhibits *S. aureus* biofilm formation and potentiates antibiotic activity against biofilm-related infections [21]. In light of previous studies, our work constitutes a comprehensive investigation into the potential of extracts derived from *V. vinifera* L., *C. sinensis* L., *O. europaea* L, *Q. robur* L., and *C. arabica* L. as novel natural agents against *S. aureus* biofilm infections. Our primary objectives encompass assessing the antibacterial efficacy of the previously cited plant extracts against both ATCC reference strains and clinically isolated *S. aureus* strains, with a specific focus on their ability to inhibit biofilm formation. Indeed, while several studies have investigated the antibacterial properties of plant extracts, significant gaps remain regarding their specific antibiofilm activities, particularly against clinically isolated *S. aureus* strains, which exhibit a high degree of variability in biofilm formation and antibiotic resistance. Employing standardized in vitro assays, we conducted rigorous evaluations of extract potency. The anticipated results include the identification of promising plant-derived compounds capable of effectively disrupting *S. aureus* biofilms, thus offering new avenues for combating antibiotic-resistant infections. Moreover, by filling existing knowledge gaps regarding the efficacy of plant extracts against *S. aureus* biofilms, this study contributes to the development of alternative prevention and treatment strategies. The significance of this research lies in its potential to address the escalating threat of antibiotic resistance while paving the way for the utilization of natural compounds as adjunctive therapies.

## 2. Results

### 2.1. In Vitro Antibacterial Activity of the Plant-Derived Extracts Against Staphylococcus aureus Strains

The antibacterial activity of the selected commercial plant extracts was initially evaluated using the agar diffusion method [22,23]. This method allows for a preliminary screening of antimicrobial agents by measuring the inhibition zones formed around impregnated disks. The diameters of the inhibition zones generated by the tested extracts or cefepime against *S. aureus* strains are reported in Table 1. All the extracts exhibited growth inhibition activity against the strains tested in this study. The most promising effect was observed against both the ATCC 29213 strain and the clinically isolated strain IG22. In particular, extract 5 (*Q. robur* wood dry extract) and the three *V. vinifera* L. extracts, 1, 4, and 7, showed promising effects as, in many cases, they were comparable to the reference antibiotic cefepime, since the determined inhibition zone diameter ranges were found to be 8–26, 12–23, and 6–22 mm, respectively. These findings suggest that the plant extracts analyzed possess notable antibacterial properties against both reference and clinically relevant strains of *S. aureus*.

Following these initial findings, the minimum inhibitory concentration (MIC) was determined for each plant extract to further quantify their antibacterial potency in accordance with Clinical Laboratory Standards Institute (CLSI) recommendations [24]. This evaluation was conducted using the broth microdilution method. The antibacterial activity observed in all the extracts demonstrates a shared efficacy against the pathogenic strains with moderate differences. The results are reported in Table 2. Here, again, the most susceptible strain turned out to be *S. aureus* IG22, against which extracts 1, 4, and 5 were particularly effective, displaying MIC values of 0.2 mg/mL. Similarly, the results indicated that even the ATCC 6538 strain was susceptible to the action of these extracts. Specifically, extracts 4 and 5 exhibited an MIC of 0.2 mg/mL, while extracts 16 and 7 showed an MIC of 0.4 mg/mL. Furthermore, noteworthy activity was detected for extract 5, which demonstrated MIC values ranging from 0.2 to 12.4 mg/mL. Of special interest is the action against the methicillin-resistant *S. aureus* strain ATCC 43300, for which an MIC of 0.4 mg/mL was calculated. The efficacy of the three *V. vinifera* L. extracts (1, 4, and 7) was noteworthy and comparable, with MIC values ranging from 0.2 to 12.4 mg/mL, 0.2 to 6.2 mg/mL, and 0.4 to 12.4 mg/mL, respectively. In contrast, extracts 2 and 3 were less effective overall, with MICs in the range of 3.1 to 12.4 mg/mL. On the contrary, extract 6 also showed strong efficacy, with MICs ranging from 0.4 to 6.2 mg/mL, including an MIC of 0.8 mg/mL against the ATCC 43300 strain.

### 2.2. Antibiofilm Activity

The encouraging results obtained through the MIC test prompted us to investigate the potential inhibitory effect of these extracts on the biofilm growth of the *S. aureus* strains considered in this study. The evaluation of the antibiofilm effect was made by means of the XTT assay. Extracts 1–7 were tested at concentrations of 32, 64, and 128 mg/mL, showing a concentration-dependent trend in their antibiofilm activity. The graphs illustrating the percentage of biofilm growth for each strain at different extract concentrations are provided in the Supplementary Materials (Figures S1–S7). The results, presented in Table 3, are expressed as percentages of biofilm growth relative to untreated controls (100% biofilm growth). It is noteworthy that for the comparison between the extracts, Table 3 reports only the results for the concentration of 128 mg/mL, which has been selected as the concentration showing the highest efficacy. Notably, all the extracts tested at this concentration demonstrated significant activity in inhibiting biofilm formation across the different strains. Notably, extract 6 (*C. arabica* L. seed dry extract) showed particularly impressive results, reducing the biofilm growth in the examined strains to a range of 8.6–24.5%. Following this, extract 3 (*O. europaea* L. fruit dry extract) also significantly affected biofilm formation, resulting in biofilm growth percentages ranging from 8.7 to 28.4%. Regarding the three *V. vinifera* L. extracts (extracts 1, 4, and 7), which were obtained using different extraction methods, the activity against the strains did not show significant variation. Specifically, the biofilm growth of the *S. aureus* strains after treatment with extract 1 (10–15:1; water) ranged from 11.4% to 21.4%, after exposure to extract 9 (drug-to-extract ratio 30–70:1; acetone/water), it ranged from 12.6% to 23.5%, and following treatment with extract 7 (drug-to-extract ratio 50–80:1; acetone/water), it ranged from 12.5% to 17.5%. These findings suggest that the extraction method may not significantly impact the antibiofilm efficacy of *V. vinifera* L. extracts. Overall, the extracts not only inhibited bacterial growth but also effectively impaired biofilm development, highlighting their importance in combating bacterial infections associated with biofilm formation.

## 3. Discussion

The increasing prevalence of antimicrobial resistance poses a significant challenge in the treatment of bacterial infections, particularly those caused by biofilm-forming strains such as *S. aureus* [25,26]. This growing resistance complicates the management of hospital-acquired infections, where *S. aureus* is a common culprit [27]. One of the primary factors contributing to the persistence of *S. aureus* infections is its ability to form biofilms. Biofilm formation gives bacteria enhanced resistance to antibiotics and host immune responses, making infections significantly harder to treat. In healthcare settings, biofilm-related infections, particularly those associated with indwelling medical devices, lead to prolonged hospital stays, higher healthcare costs, and increased mortality rates [28].

Given these challenges, there is an urgent need for novel therapeutic strategies to combat biofilm-associated infections, especially those caused by antibiotic-resistant strains like methicillin-resistant *S. aureus* (MRSA) [29]. One promising avenue for addressing this issue is the use of compounds of natural origin, such as essential oils, plant extracts, and other bioactive phytochemicals [30,31,32,33]. These natural products offer several advantages, including their ability to target bacterial growth and biofilm formation through multiple mechanisms, potentially reducing the likelihood of resistance development. Furthermore, natural extracts often exhibit low toxicity, making them safer alternatives or adjuncts to traditional antibiotic therapies [34]. Our findings are consistent with the existing scientific literature, which supports the efficacy of plant-derived compounds in inhibiting biofilm development and bacterial growth. Numerous studies have reported that various botanical extracts possess similar antimicrobial properties, reinforcing the concept that natural products could serve as a promising alternative or adjunct to traditional antibiotics. By leveraging these natural extracts, we may be able to develop novel therapeutic options that not only target planktonic bacteria but also disrupt biofilm integrity, thereby enhancing the overall effectiveness of treatment regimes in clinical settings.

For instance, *V. vinifera* L. (grape seed extract) is rich in proanthocyanidins, catechins, and epicatechins, which have been shown to exhibit significant antibacterial activity against *S. aureus*, primarily through the inhibition of cell wall synthesis and biofilm formation. According to the literature, proanthocyanidins destabilize bacterial membranes, causing increased permeability and cell lysis [35]. Moreover, proanthocyanidins affect the expression of genes related to biofilm formation and maintenance, further inhibiting the ability of bacteria like *S. aureus* to form robust biofilms [36]. It has also been reported that proanthocyanidins can decrease the stability of bacterial cell membranes, particularly in methicillin-resistant *S. aureus* (MRSA), by suppressing β-lactamase activity and reducing tolerance to osmotic pressure and ionic strength, thereby enhancing the effectiveness of β-lactam antibiotics [37]. Catechins and epicatechins disrupt biofilm formation by interfering with quorum sensing and inhibiting EPS production [38,39]. Our results are in line with those reported in the literature, which highlighted the antibacterial activity of *V. vinifera* L. extracts against various strains of *S. aureus*, including methicillin-resistant *S. aureus* (MRSA), with MIC values varying across the studies [15,40]. Indeed, our results demonstrated the promising antibacterial efficacy of the three *V. vinifera* L. (1, 4, and 7) extracts considered against the *S. aureus* strains tested in this study, with the MIC values ranging from 0.2 to 15.5 mg/mL. However, despite being obtained through different extraction methods (drug-to-extract ratio 10–15:1 water; 30–70:1 acetone/water, and 50–80:1 acetone/water), no microbiologically significant differences were observed among the three extracts in terms of activity. Additionally, we observed notable antibiofilm effects, achieving up to 90% eradication of biofilms, further supporting the potential of *V. vinifera* L. extracts in combating biofilm-related infections. Similarly, *C. sinensis* L. (green tea) contains various polyphenols, particularly epigallocatechin gallate (EGCG), which has demonstrated effectiveness in inhibiting the biofilm development of *S. aureus*. Several studies report that EGCG not only reduces bacterial viability but also interferes with biofilm-associated exopolysaccharide production, leading to enhanced susceptibility of biofilm-forming strains to conventional antibiotics [41,42]. In addition, EGCG has been shown to increase intracellular reactive oxygen species (ROS) levels, leading to oxidative stress in bacteria. This oxidative stress disrupts bacterial cellular functions and inhibits their growth [43]. EGCG can cause physical damage to bacterial cell membranes, leading to morphological changes and cell death [44]. Although *C. sinensis* L. aqueous leaf dry (extract 2) did not perform as well as some of the other extracts in our study, with MIC values ranging between 3.1 and 12.4 mg/mL, it also demonstrated promising antibiofilm efficacy, showing biofilm growth inhibition between 70% and 80%. These findings are in line with those previously reported. Indeed, *C. sinensis* L. has demonstrated antibacterial activity against *S. aureus*, with MIC values ranging from 4 to 16 mg/mL [45]. This suggests that despite its relatively higher MIC, *C. sinensis* L. remains a valuable candidate for further exploration as a natural antibiofilm agent.

*O. europea* L. (olive) extract activity could be primarily attributed to the presence of hydroxytyrosol (and its derivatives) and verbascoside. Hydroxytyrosol, a potent antioxidant, disrupts bacterial membranes and inhibits oxidative stress responses. Moreover, hydroxytyrosol acetate also interacts with bacterial DNA, enhancing the fluorescent intensity of DNA-binding molecules and mediating supercoiled DNA relaxation. This interaction suggests a dual mechanism of action, targeting both the bacterial membrane and genetic material [46]. Verbascoside further contributes by targeting bacterial adhesion proteins and EPS synthesis. Indeed, in *S. aureus,* verbascoside affects protein synthesis by inhibiting leucine incorporation, which is crucial for bacterial growth and survival [47]. In addition, its ability to act as a dual inhibitor of the serine/threonine phosphatase Stp1 in *S. aureus* has been reported, utilizing both competitive and allosteric mechanisms [48]. This inhibition affects the regulation of virulence factors, reducing the bacteria’s ability to cause disease. These mechanisms are consistent with the observed efficacy, indicating that *O. europaea* L. extracts can effectively combat *S. aureus* biofilms. Our results highlighted that extract 3 demonstrated notable antibacterial and antibiofilm activity, with MICs ranging from 0.4 to 12.4 mg/mL and biofilm growth decreases of 8.7–28.4%. *O. europea* L. extract from various cultivars with inhibition percentages ranging from 83% to 93% [49]. The antimicrobial activity of *O. europaea* L. extract is attributed to its ability to damage microbial membranes and inhibit efflux pump activity, which are crucial for biofilm development and maintenance. Furthermore, olive leaf extracts have been shown to modulate quorum sensing genes, which are essential for biofilm formation and virulence in bacteria, thereby reducing biofilm formation and virulence factor production [50,51]. *Q. robur* (oak bark) has been noted for its high tannin content, which has demonstrated antibacterial activity against *S. aureus* by precipitating microbial proteins and impairing cell membrane integrity. Studies have shown that oak bark extracts can disrupt biofilm formation of various bacterial strains by modulating the expression of virulence genes in bacteria. Multiple mechanisms are involved in the antimicrobial activity of tannins, the most studied being the interaction with surface proteins, the damage to the activity of lipid bilayer membranes or cell walls, and the ability to interfere with some metallic ions (especially iron) that act as cofactors for key enzyme-dependent processes [52,53]. The most important antibacterial compounds belonging to the tannins group previously reported in *Quercus* bark extracts are gallic acid, ellagic acid, vescalagin, and castalagin [20]. Our results indicated a marked activity of extract 5, even below mg/mL in many cases. The effect found on planktonic cells was confirmed by the antibiofilm action observed, which highlights the potential for utilizing extract 5 as a powerful agent in combating biofilm-associated infections. Studies have shown that extracts from the bark of *Q. robur* exhibit significant antibacterial activity. The extracts, particularly those prepared with 70% acetone, have demonstrated effectiveness against *S. aureus* and *Escherichia coli* [10]. Additionally, water–ethanolic extracts with high ethanol concentrations (60%, 70%, and 96%) have shown strong antimicrobial effects against *Pseudomonas aeruginosa* and *Candida albicans* [54]. The leaves of *Q. robur* also possess antimicrobial properties. Methanolic and aqueous extracts have been effective against both Gram-positive and Gram-negative bacteria, including *S. aureus*, with MIC values around 10 mg/mL [55].

Lastly, *C. arabica* L. (coffee) extracts contain several bioactive compounds, including chlorogenic acids and caffeine, which exhibit selective antibacterial properties. Research has shown that these compounds can inhibit *S. aureus* biofilm formation while reducing pathogenicity by affecting bacterial metabolic processes [56]. The results of this study confirm the remarkable antibacterial and antibiofilm activity of *C. arabica* L. extract (6), which reduced biofilm growth to as low as 8.6–24.5%, positioning it among the most effective extracts tested. Its MIC values, ranging from 0.4 to 12.4 mg/mL, highlight its strong antimicrobial potential. Notably, the elevated levels of chlorogenic acid in this extract may play a pivotal role in its potent antibiofilm activity. Chlorogenic acid exerts its effects by disrupting bacterial cell walls, interfering with the synthesis of exopolysaccharides (EPS), and reducing the structural integrity of the biofilm matrix [57]. Additionally, chlorogenic acid also acts as a quorum sensing inhibitor, interfering with signaling pathways that regulate biofilm formation [58].

Caffeine, another principal component of *C. arabica* L. extracts, complements the action of chlorogenic acid by affecting bacterial metabolic processes and inhibiting quorum sensing pathways [59].

The ability of *C. arabica* extract to disrupt biofilm integrity is particularly noteworthy, as biofilms are notoriously difficult to eradicate and are often associated with persistent infections. Compared to findings in the literature, our results further validate the role of *C. arabica* L. as a natural source of bioactive compounds with significant antibacterial and antibiofilm effects.

Overall, our findings echo the growing body of evidence suggesting that the incorporation of plant extracts in antimicrobial treatments could not only augment the effectiveness of traditional antibiotics but also help circumvent the challenges posed by resistance mechanisms, thus providing valuable insights into novel combinatory therapies.

## 4. Material and Methods

### 4.1. Materials

#### 4.1.1. Plant Extract

The commercial dry extracts, including *Vitis vinifera* L. seed dry extract (10–15:1; water) (1), *Camellia sinensis* L. aqueous leaf dry extract (2), *Olea europaea* L. fruit dry extract (3), *Vitis vinifera* L. seed dry extract (drug-to-extract ratio 30–70:1; acetone/water) (4), *Quercus robur* wood dry extract (5), *Coffea arabica* L. seed dry extract (6), and *Vitis vinifera* L. seed dry extract (drug-to-extract ratio 50–80:1; acetone/water) (7), were kindly provided by Indena SpA (Milan, Italy). The details of the commercial plant dry extracts are reported and summarized in Table 4.

*V. vinifera* L. grape seed dry aqueous extract (1): The total phenolic content (proanthocyanidins) was 97.7% *w*/*w* dry extract, determined using spectrophotometric analysis (Folin method). The HPLC-UV analysis reported the presence of catechin monomers (sum of catechin and epicatechin) at 6.8% *w*/*w* dry extract. Proanthocyanidin dimers, expressed as catechin, comprised 7.6% *w*/*w* dry extract. *C. sinensis* L. or green tea leaves dry aqueous extract (2): The total phenolic content was 42.6% *w*/*w* dry extract, determined using spectrophotometric analysis (Folin method). HPLC-UV analysis reported the presence of total catechins, expressed as epigallocatechin-3-O-gallate, at 18.9% *w*/*w* dry extract, of which 8.7% *w*/*w* dry extract was epigallocatechin-3-O-gallate. Caffeine content was 5.8% *w*/*w* dry extract. *O. europaea* L. olive fruit dry hydroalcoholic extract (3): The total phenolic content was 11.8% *w*/*w* dry extract, determined using spectrophotometric analysis. HPLC-UV analysis reported verbascoside at 2.4% *w*/*w* dry extract and hydroxytyrosol and its derivatives at 5% *w*/*w* dry extract. *V. vinifera* L. grape seed dry acetone/water (30:70) extract (4): HPLC-UV analysis reported the presence of catechin (sum of catechin and epicatechin) at 15.1% *w*/*w* dry extract. *Q. robur* L. oak dry extract (5): the total polyphenol content was 75.1% *w*/*w* dry extract, determined using spectrophotometric analysis. *C. arabica* L. green coffee seed dry hydroalcoholic extract (6): HPLC-UV analysis reported the presence of caffeoylquinic acids, expressed as chlorogenic acid, at 25.2% *w*/*w* dry extract, with chlorogenic acid representing 15% *w*/*w*. Caffeine content was 5.2% *w*/*w* dry extract, and trigonelline content was 2.6% *w*/*w* dry extract. *V. vinifera* L. grape seed dry acetone/water extract (7): HPLC-UV analysis reported the presence of catechin (sum of catechin and epicatechin) at 21.6% *w*/*w* dry extract. Proanthocyanidins (dimers) were 24.2% *w*/*w* dry extract. All chemical and data analyses reported here were performed by the manufacturer.

#### 4.1.2. Bacterial Strains

In this study, seven bacterial strains from American Type Culture Collection (ATCC, Rockville, MD, USA) and clinical isolates were used for the tests: *Staphylococcus aureus* ATCC 29213, *Staphylococcus aureus* ATCC 6538, *Staphylococcus aureus* ATCC 25923, *Staphylococcus aureus* 43300, and clinical isolates *Staphylococcus aureus* Ig22, *Staphylococcus aureus* Ig23, and *Staphylococcus aureus* Ig24. Table 5 reports the isolation sources and resistance profiles of each strain. All the clinical isolates were furnished by the Interdisciplinary Department of Medicine, University of Bari “Aldo Moro”, Piazza G. Cesare 11, 70124, Bari, Italy.

#### 4.1.3. Medium and Culture Conditions

The culture media used in this study are Mueller–Hinton Broth (Oxoid S.p.A., Rodano, Milano, Italy) and Mueller–Hinton Agar (Oxoid S.p.A., Rodano, Milano Italy). Mueller–Hinton Broth (MHB) is a non-selective medium, recommended by CLSI (Clinical and Laboratory Standards Institute) to establish the minimum inhibitory concentration (MIC); MHA plates are used to seed bacterial microorganisms. For studies of the antibiofilm activity of the extracts, Tryptic Soy Broth (TSB + 2% glucose, Oxoid S.p.A., Rodano, Milano Italy) was used as culture medium. Colonies were recognized with Api system and were typically smooth, raised, translucent, and circular, up to 6–8 mm in diameter, and usually yellow/gold to orange in color. Strains were preserved frozen in cryovials at a temperature below 4 °C to minimize mutations that strains may incur and to preserve morphological and biochemical characteristics of the different strains.

Petri dishes containing solid media are sealed with parafilm, a polyolefin and paraffin wax, as soon as they are ready. They are then stored upside down in a fresh and dry place to preserve any changes in the product. Powder media in plastic containers should be stored in a dry environment. Liquid media are stored in a dark and dry environment after sterilization to ensure perfect product integrity.

### 4.2. Methods

#### 4.2.1. Preparations of the Test Solutions

For antibacterial testing, each extract was dissolved in sterile water to obtain a stock solution. All the extracts that were part of our experiment were subjected to microbial contamination control. The result confirmed that all products showed no presence of bacteria or microbial contaminants.

#### 4.2.2. Antibacterial Studies

The agar disk diffusion method was employed for preliminary screening of the antibacterial activity of the extracts. The assays were performed according to the methodology recommended by the Clinical and Laboratory Standard Institute (CLSI) and M-44A protocol [23]. Briefly, Petri dishes with MHA were inoculated with liquid bacterial suspensions. The turbidity of the bacterial cell suspension was calibrated to 0.5 McFarland Standard using a spectrophotometric approach (OD_625_ nm 0.08–0.10). Sterilized paper disks were placed on agar plates, and 10 μL of antimicrobial solution (final concentration of each extract amounting to 20 mg/mL) was poured on top of the disks. The plates were then incubated at 37 °C for 24 h. Subsequently, the zone of inhibition of antimicrobial activity was evaluated. After conducting a qualitative analysis of the activity of the extracts, the broth microdilution method was used to determine the in vitro minimum inhibitory concentrations (MICs, mg/mL), Following the recommendations of the CLSI (protocol M7A9) [24]. The concentration of the extracts investigated was set to its highest value to create stock solutions (248 mg/mL). Then, the stock solutions were diluted 1:10 with Cation-Adjusted Mueller–Hinton Broth (Oxoid, Milan, Italy). After that, a series of concentrations were obtained in the wells by performing two-fold serial dilutions in the appropriate test medium. Even in this case, the turbidity of the bacterial cell suspension was calibrated to 0.5 McFarland Standard. The standardized suspension was then further diluted (1:100) with MHB to reach 1–2 × 10^6^ CFU/mL. A 200 µL aliquot of the final inoculum was inserted into each well. As a control for growth (negative control), several wells containing only inoculated broth were prepared. The plates were incubated at 37 °C for 24 h, and the MIC values were calculated as the lowest concentration of compounds without visible bacterial growth. In wells containing only culture medium (negative control), no bacterial growth was detected. Each experiment was carried out in duplicate and repeated three times to determine the MICs. Cefepime served as positive control.

#### 4.2.3. Biofilm Biomass Measurement and Reduction

Before assessing the antibiofilm activity of the extracts under investigation, we conducted preliminary crystal violet staining to verify the ability of strains to form a biofilm, following the procedure described by Stepanović with some modifications [60]. Briefly, biofilms attached to wells were fixed with 250 μL of methanol 98% for 30 min. We emptied the wells and retrieved residuals, then we let them dry for 20 min. Fixed biofilms were dyed with 200 μL of crystal violet for 10 min. The exceeding pigment was removed by washing the plate with distilled water. Then, the plates were left to dry for 45 min. The residual was suspended again with glacial acetic acid 33%, at 200 μL per well for 20 min. Optical density at 570 nm was then determined with a microplate reader.

To assess the antibiofilm activity of the extracts, we conducted, in vitro, the colorimetric XTT *(2,3-bis(2-methoxy-4-nitro-5-sulphophenyl)-5-[(phenylamino)carbonyl]-2H-tetrazolium hydroxide)* assay. Living bacterial cells can reduce tetrazolium salt in a hydro soluble composite called formazan, owing to NADH-mitochondrial dehydrogenases of the electron transport system (ETS) using an artificial electron acceptor (redox pigment) that competes with oxygen [61]. Briefly, 200 μL of bacterial culture (10^6^ CFU/mL) was added to each well of a 96-well flat microtiter plate. The plate was initially incubated for 4 h at 37 °C on a rocker table to facilitate uniform cell distribution. Subsequently, the plate was incubated under static conditions for an additional 18 h at 37 °C to allow cell attachment and biofilm formation. Then, 200 μL of each extract, prepared at concentrations of 32, 64, or 128 mg/mL, was added to the test wells. As a positive control, the reference antibiotic cefepime (Sigma-Aldrich S.p.a., Milan, Italy) was used, while the negative control consisted only of the culture medium without any extract or antibiotics.

After incubation, the contents of each well were aspirated, and the wells were washed with 100 μL of sterile Phosphate-Buffered Saline adjusted at a pH of 7.4. After the preparation, sterilization in an autoclave at 120 °C was performed for 15 min. The solution was preserved at 4 °C. Washing with PBS was necessary to remove loosely adherent and nonviable cells. Following incubation, 200 μL of each extract was added to the wells at concentrations of 32, 64, and 128 mg/mL. Following incubation, the medium was aspirated from each well, and the supports were washed with 100 µL of PBS each. A volume of 200 µL of a solution formed by PBS with the addition of XTT solution (1 mg/mL) and Menadione (0.4 mM) at a ratio of 6:1 were then added to each well. This solution was prepared by dissolving XTT in sterile water (obtained by filtration) and Menadione in dimethyl sulfoxide. The plate with the supports was incubated for 2 h at 37 °C. After 2 h, the plate was read with a microplate spectrophotometer Tecan Infinite M1000 Pro multiplate reader (Tecan, Cernusco S.N., Italy) at a 490 nm wavelength, where there was a maximum Formazan absorbance peak. The percentage of the reduction in the biofilm obtained with the combinations studied was calculated using the following formula: percentage of biofilm reduction = (OD control well−OD experimental)/(OD control well) × 100 [29]. The results are expressed as means ± SDs of at least three distinct measurements performed in triplicate.

#### 4.2.4. Statistical Analysis

All the data are presented as means ± standard deviations (SDs), with values derived from a minimum of three independent experiments. All assays were performed in triplicate to ensure reproducibility. Statistical significance (*p* < 0.0001) was assessed using one-way ANOVA followed by Dunnett’s post hoc test in GraphPad Prism 9.0. Statistical significance was defined as a *p*-value ≤ 0.05. The corresponding graphs illustrating the results are provided in the Supplementary Materials for further reference.

## 5. Conclusions

The current issue of antimicrobial resistance, particularly in biofilm-associated infections caused by *S. aureus*, necessitates innovative approaches for treatment. Investigations into the antibacterial and antibiofilm inhibitory activities of plant extracts provide valuable insights into effective strategies that may significantly improve patient outcomes in the face of increasing resistance threats. This study investigated the antibacterial and antibiofilm activities of plant extracts derived from *V. vinifera* L., *C. sinensis* L., *O. europaea* L, *Q. robur* L., and *C. arabica* L. against *S. aureus* strains derived both from the ATCC collection and clinical isolates. The interesting results obtained in this study showed that *Q. robur* possessed the most potent antibacterial effects, as evidenced by its low MIC values (often below 1 mg/mL), whereas *C. arabica* demonstrated exceptional antibiofilm efficacy, reducing biofilm growth by up to 91.4%. These findings underscore the significant potential of these natural extracts, as it is rare to identify compounds of natural origin with such pronounced activities. These results can provide a starting point for further studies aimed at exploring the potential of these natural compounds as effective agents against *S. aureus* infections. In future studies, these plant extracts could be investigated for their potential synergistic effects with conventional antibiotics, which may help to address the issue of antibiotic resistance and allow for the use of lower antibiotic doses. As we advance, incorporating these plant-based solutions into formulations can, in turn, be used in clinical practice and may not only alleviate the burden of resistant infections but also promote a more sustainable approach to healthcare by decreasing the reliance on synthetic antimicrobials.

## Figures and Tables

**Table 1:** Antibacterial activity of plant extracts and cefepime measured as diameter of inhibition zone (mm) against ATCC and clinical isolates of *Staphylococcus aureus*.

*Staphylococcus aureus* Strains	1	2	3	4	5	6	7	Cefepime
IG22	25.0 ± 1.3	14.0 ± 1.4	14.0 ± 1.4	23.0 ± 1.3	25.0 ± 1.4	12.0 ± 1.0	12.0 ± 1.0	27.0 ± 1.2
IG23	26.0 ± 1.4	17.0 ± 1.1	17.0 ± 0.9	13.0 ± 1.1	24.0 ± 1.1	14.0 ± 1.3	14.0 ± 1.4	25.0 ± 1.3
IG24	8.0 ± 1.0	9.0 ± 0.19	9.0 ± 1.2	13.0 ± 0.6	6.0 ± 0.8	6.0 ± 0.7	6.0 ± 0.6	23.0 ± 1.1
ATCC 25923	15.0 ± 1.3	14.0 ± 1.3	15.0 ± 0.8	16.0 ± 0.8	14.0 ± 0.5	15.0 ± 1.3	14.0 ± 0.7	24.0 ± 0.5
ATCC 29213	26.0 ± 1.2	13.0 ± 1.3	15.0 ± 1.3	15.0 ± 1.3	23.0 ± 1.0	25.0 ± 1.2	22.0 ± 1.0	24.0 ± 0.4
ATCC 43300	4.0 ± 0.3	13.0 ± 1.3	18.0 ± 0.9	15.0 ± 1.3	27.0 ± 1.4	19.0 ± 1.1	14.0 ± 1.4	26.0 ± 0.7
ATCC 6538	10.0 ± 1.3	10.0 ± 0.3	10.0 ± 0.1	12.0 ± 0.5	18.0 ± 0.9	7.0 ± 0.9	20.0 ± 1.2	28.0 ± 1.0

Values are means of three experiments performed in duplicate (mm ± SD). Sample deposit: 10 µL, final concentration of each extract amounting to 20 mg/mL. Control: cefepime 30 μg/disk. The diameter of the disk (8 mm) is not included. 1: *Vitis vinifera* L. seed dry extract (10–15:1; water); 2: *Camellia sinensis* aqueous leaf dry extract; 3: *Olea europea* fruit dry extract; 4: *Vitis vinifera* L. seed dry extract (drug-to-extract ratio 30–70:1; acetone/water); 5: *Quercus robur* wood dry extract; 6: *Coffea arabica* L. seed dry extract; 7: *Vitis vinifera* L. seed dry extract (drug-to-extract ratio 50–80:1; acetone/water).

**Table 2:** Minimum inhibitory concentrations of plant extracts (MIC, mg/mL) and cefepime (MIC, µg/mL) required to inhibit the growth of *Staphylococcus aureus* strains.

*Staphylococcus aureus* Strains	1	2	3	4	5	6	7	Cefepime
IG22	0.2	3.1	6.2	0.2	0.2	0.8	0.8	2.0
IG23	12.4	12.4	12.4	6.2	12.4	6.2	3.1	4.0
IG24	6.2	12.4	6.2	0.8	3.1	6.2	6.2	1.0
ATCC 25923	3.1	3.1	3.1	0.4	0.4	0.8	0.4	2.0
ATCC 29213	6.2	12.4	6.2	0.8	3.1	6.2	6.2	4.0
ATCC 43300	12.4	12.4	6.2	0.4	0.4	0.8	12.4	2.0
ATCC 6538	12.4	12.4	6.2	0.2	0.2	0.4	0.4	2.0

1: *Vitis vinifera* L. seed dry extract (10–15:1; water); 2: *Camellia sinensis* L. aqueous leaf dry extract; 3: *Olea europea* fruit dry extract; 4: *Vitis vinifera* L. seed dry extract (drug-to-extract ratio 30–70:1; acetone/water); 5: *Quercus robur* wood dry extract; 6: *Coffea arabica* L. seed dry extract; 7: *Vitis vinifera* L. seed dry extract (drug-to-extract ratio 50–80:1; acetone/water).

**Table 3:** Effect of plant extracts on *Staphylococcus aureus* biofilm formation measured by XTT assay.

*Staphylococcus aureus* Strains	1	2	3	4	5	6	7	Cefepime
IG22	21.4 ± 1.2	20.5 ± 0.8	15.7 ± 0.9	15.7 ± 0.6	23.6 ± 1.3	18.5 ± 1.3	15.3 ± 1.3	0.3 ± 1.2
IG23	11.4 ± 0.7	19.9 ± 0.6	16.1 ± 0.4	23.5 ± 0.8	15.1 ± 08	14.3 ± 0.6	13.4 ± 0.3	0.5 ± 0.7
IG24	15.7 ± 11	33.1 ± 0.9	15.0 ± 0.9	14.6 ± 10	27.2 ± 0.9	11.8 ± 0.8	17.1 ± 0.9	1.5 ± 1.1
ATCC 25923	15.6 ± 1.0	18.6 ± 13.0	28.4 ± 0.9	12.6 ± 1.0	20.3 ± 1.2	24.5 ± 0.9	12.5 ± 1.1	1.2 ± 1.0
ATCC 29213	17.1 ± 0.7	34.4 ± 0.3	13.2 ± 0.7	19.7 ± 1.0	21.3 ± 039	15.1 ± 0.7	13.4 ± 0.7	1.6 ± 0.7
ATCC 43300	16.4 ± 0.5	18.9 ± 0.9	14.5 ± 0.8	19.5 ± 0.6	23.5 ± 1.1	14.3 ± 1.0	17.5 ± 0.8	1.4 ± 0.5
ATCC 6538	18.3 ± 0.5	33.8 ± 0.5	8.7 ± 1.1	15.5 ± 0.9	24.6 ± 06	8.6 ± 0.6	12.7 ± 0.6	1.3 ± 0.5

1: *Vitis vinifera* L. seed dry extract (10–15:1; water); 2: *Camellia sinensis* L. aqueous leaf dry extract; 3: *Olea europea* fruit dry extract; 4: *Vitis vinifera* L. seed dry extract (drug-to-extract ratio 30–70:1; acetone/water); 5: *Quercus robur* wood dry extract; 6: *Coffea arabica* L. seed dry extract; 7: *Vitis vinifera* L. seed dry extract (drug-to-extract ratio 50–80:1; acetone/water). Values are means of three experiments performed in duplicate, indicated as percentage growth of bacterial biofilm ± SD. Concentration of each extract used: 128 mg/mL. Reference antibiotic: cefepime, 20 µg/mL.

**Table 4:** Details of plant extracts and their main constituents, as provided by the manufacturer.

n.	Plant Name (Botanical Family)	Batch Number	Part Plant	Extraction Solvent	Drug-to-Extract Ratio	Total Phenolic Content (TPC) % *w*/*w* Dry Extract	HPLC-UV Analysis % *w*/*w* Dry Extract
(1)	*Vitis vinifera* L. (Vitaceae) Grape	16,335	Seeds	H_2_O	10–15:1	97.7	Catechin + epicatechin = 6.8 Proanthocyanidins = 7.6
(2)	*Camellia sinensis* L. (Theaceae) Green tea	16,092	Leaves	H_2_O	2–3:1	42.6	Total catechins = 18.9 Epigallocatechin-3-O-gallate = 8.7 Caffeine = 5.8
(3)	*Olea europaea* (Oleaceae) Olive	16,319	Fruit	H_2_O-EtOH	11–14:1	11.8	Verbascoside = 2.4 Hydroxytyrosol and derivatives = 5
(4)	*Vitis vinifera* L. (Vitaceae) Grape	13,460	Seeds	H_2_O-acetone	30–70:1	n.r.	Catechin + epicatechin = 15.1
(5)	*Quercus robur* (Fagaceae) Oak	23,403	n.r.	n.r.	n.r	75.1	n.r.
(6)	*Coffea arabica* L. (Rubiaceae) Green coffee	4,048,100	Seeds	H_2_O-EtOH	8–4:1	n.r.	Total caffeoylquinic acids = 25.2 Chlorogenic acid = 15 Caffeine = 2.6
(7)	*Vitis vinifera* L. (Vitaceae) Grape	16,193	Seeds	H_2_O-acetone	50–80:1	n.r.	Catechin + epicatechin = 21.6 Proanthocyanidins = 24.2

n.r.: not reported.

**Table 5:** *Staphylococcus aureus* strains used in the study, including their isolation sources and resistance profiles. MSSA refers to methicillin-susceptible *S. aureus*, while MRSA refers to methicillin-resistant *S. aureus*.

Strain	Isolation Source	Resistance
*Staphylococcus aureus* ATCC 29213	Wound	MSSA
*Staphylococcus aureus* ATCC 25923	Clinical isolate	MSSA
*Staphylococcus aureus* ATCC 6538	Lesion	MSSA
*Staphylococcus aureus* ATCC 43300	Clinical isolate	MRSA
*Staphylococcus aureus* Ig22	Blood	MRSA
*Staphylococcus aureus* Ig23	Urine	MRSA
*Staphylococcus aureus* Ig24	Blood	MRSA

## Data Availability

The data are contained within the manuscript.

## Supplementary Materials

The following supporting information can be downloaded at https://www.mdpi.com/article/10.3390/microorganisms13020454/s1: Figures S1–S7: Graphs showing the percentage of biofilm growth for each *S. aureus* strain exposed to different concentrations (32, 64, and 128 mg/mL) of each plant extract. Statistical analyses are also included for each dataset. These supplementary figures provide detailed insights into the concentration-dependent effects of the extracts on biofilm inhibition, complementing the main findings reported in the manuscript.

## Abbreviations

OD (optical density); XTT (2,3,5-triphenyl tetrazolium chloride); MHB (Mueller–Hinton Broth).
