# Supplementary material for: Antibiofilm Effects of Plant Extracts Against Staphylococcus aureus
Source: Microorganisms. 2025 Feb 19;13(2):454. doi: 10.3390/microorganisms13020454 (PMC11858306; doi:10.3390/microorganisms13020454)
Supplement: Supplementary file 1 [file microorganisms-13-00454-s001.zip › microorganisms-3420840-supplementary.pdf]

Supplementary Material

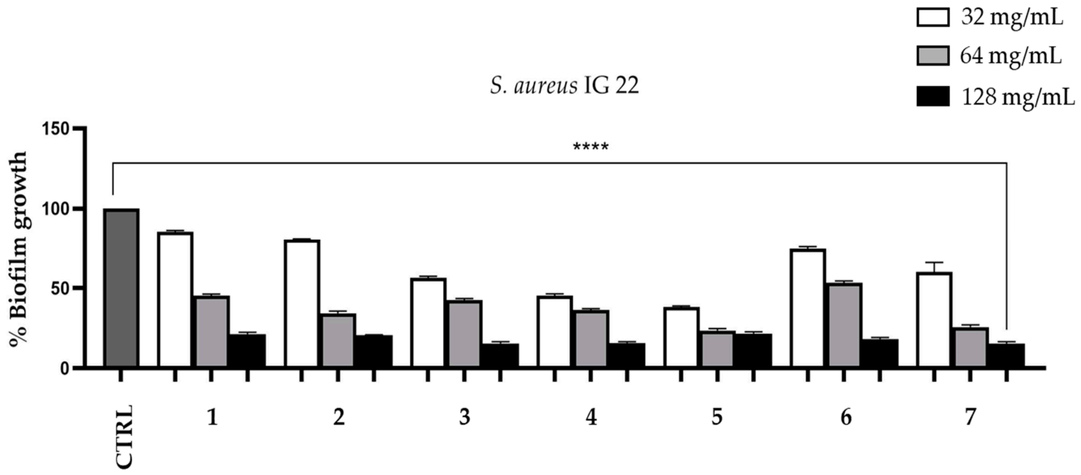

Figure S1. Percentage of biofilm growth of *Staphylococcus aureus* IG22 treated with plant extracts **1**–**7** at concentrations of 32 mg/mL, 64 mg/mL, and 128 mg/mL. The bars represent the mean ± standard deviation (SD) from three independent experiments. All data are statistically significant compared untreated control (CTRL, corresponding to the 100% of biofilm growth). Significant differences are denoted by **** $p < 0.0001$.

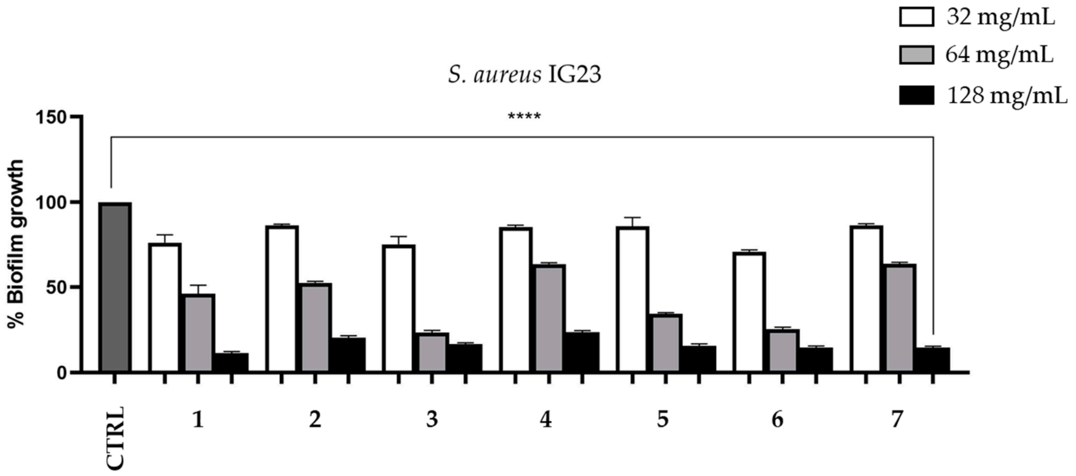

Figure S2. Percentage of biofilm growth of *Staphylococcus aureus* IG23 treated with plant extracts **1**–**7** at concentrations of 32 mg/mL, 64 mg/mL, and 128 mg/mL. The bars represent the mean ± standard deviation (SD) from three independent experiments. All data are statistically significant compared untreated control (CTRL, corresponding to the 100% of biofilm growth). Significant differences are denoted by **** $p < 0.0001$.

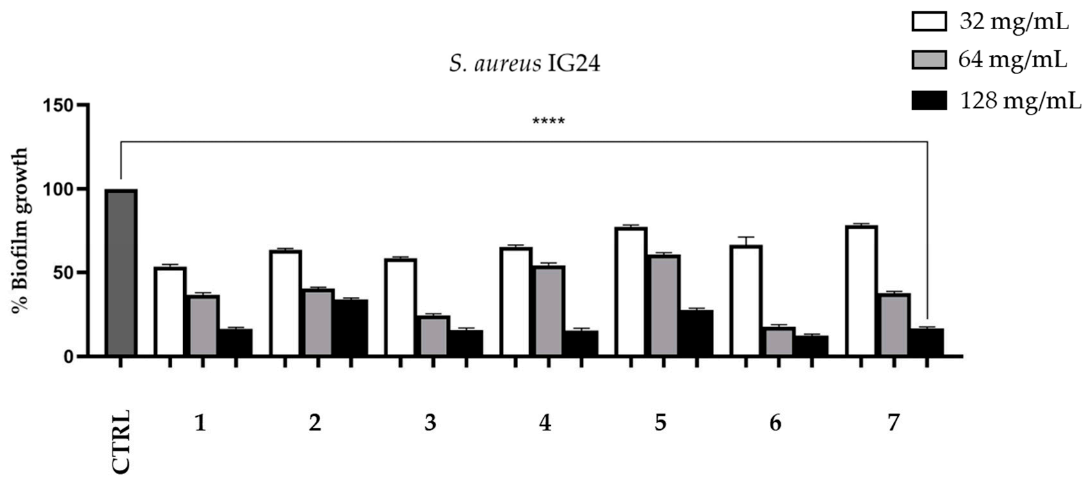

Figure S3. Percentage of biofilm growth of *Staphylococcus aureus* IG24 treated with plant extracts **1–7** at concentrations of 32 mg/mL, 64 mg/mL, and 128 mg/mL. The bars represent the mean ± standard deviation (SD) from three independent experiments. All data are statistically significant compared untreated control (CTRL, corresponding to the 100% of biofilm growth). Significant differences are denoted by **** $p < 0.0001$.

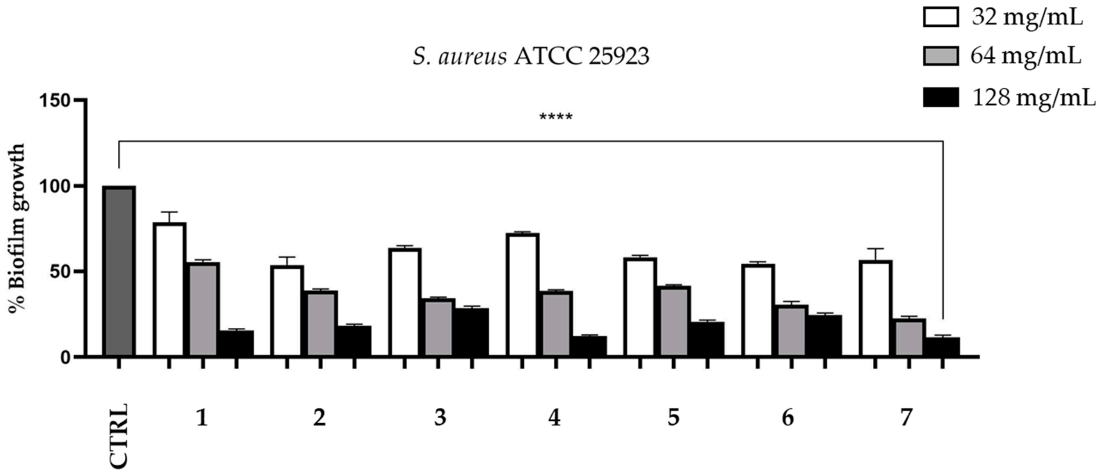

Figure S4. Percentage of biofilm growth of *Staphylococcus aureus* ATCC 25923 treated with plant extracts **1–7** at concentrations of 32 mg/mL, 64 mg/mL, and 128 mg/mL. The bars represent the mean ± standard deviation (SD) from three independent experiments. All data are statistically significant compared untreated control (CTRL, corresponding to the 100% of biofilm growth). Significant differences are denoted by **** $p < 0.0001$.

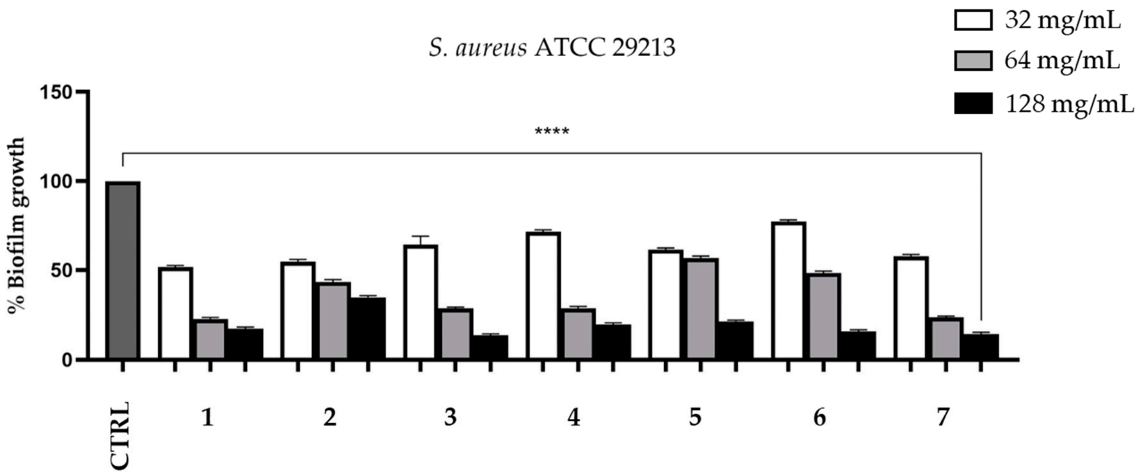

Figure S5. Percentage of biofilm growth of *Staphylococcus aureus* ATCC 29213 treated with plant extracts **1**–**7** at concentrations of 32 mg/mL, 64 mg/mL, and 128 mg/mL. The bars represent the mean ± standard deviation (SD) from three independent experiments. All data are statistically significant compared untreated control (CTRL, corresponding to the 100% of biofilm growth). Significant differences are denoted by **** $p < 0.0001$.

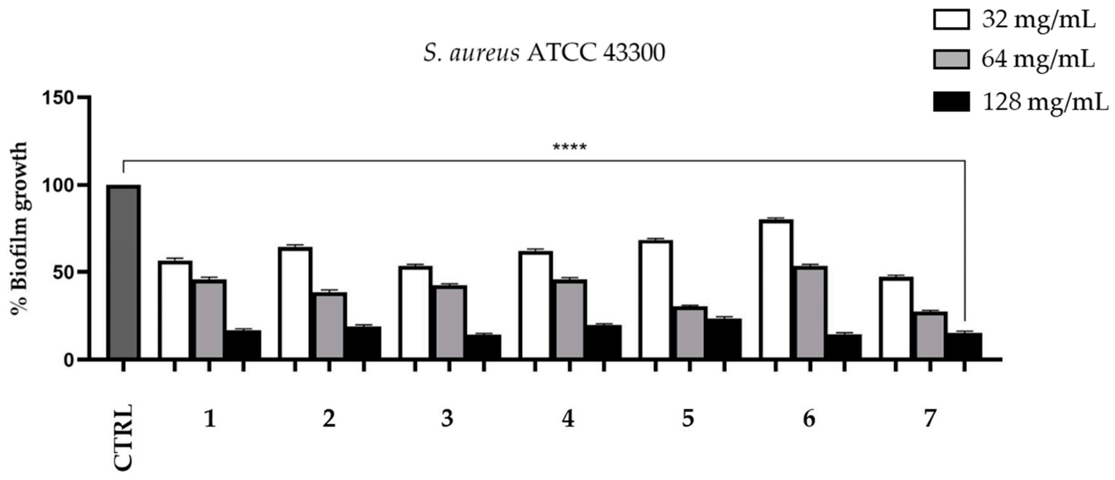

Figure S6. Percentage of biofilm growth of *Staphylococcus aureus* ATCC 43300 treated with plant extracts **1**–**7** at concentrations of 32 mg/mL, 64 mg/mL, and 128 mg/mL. The bars represent the mean ± standard deviation (SD) from three independent experiments. All data are statistically significant compared untreated control (CTRL, corresponding to the 100% of biofilm growth). Significant differences are denoted by **** $p < 0.0001$.

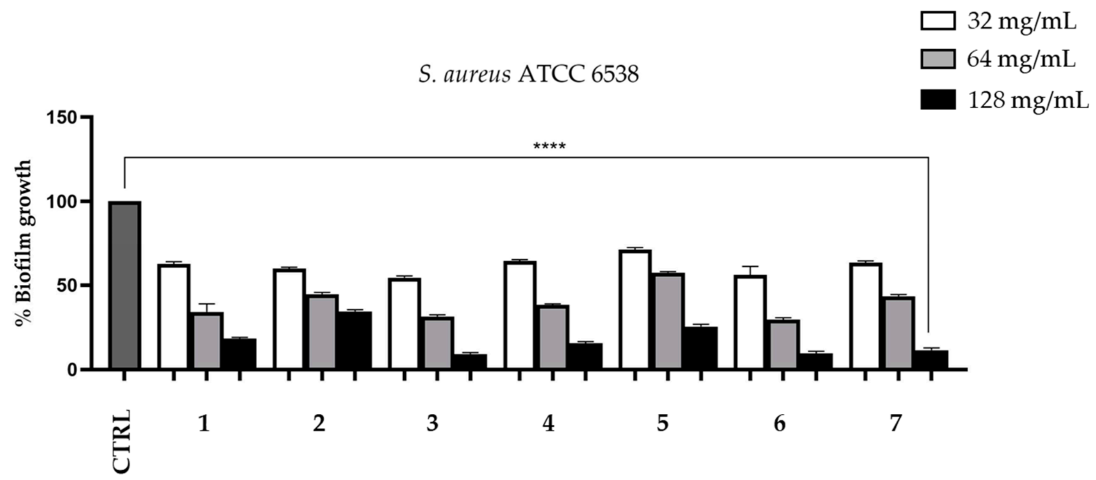

Figure S7. Percentage of biofilm growth of *Staphylococcus aureus* ATCC 6538 treated with plant extracts **1**–**7** at concentrations of 32 mg/mL, 64 mg/mL, and 128 mg/mL. The bars represent the mean ± standard deviation (SD) from three independent experiments. All data are statistically significant compared untreated control (CTRL, corresponding to the 100% of biofilm growth). Significant differences are denoted by **** $p < 0.0001$.

Statistical Analysis

All the data are presented as mean ± standard deviation (SD), with values derived from a minimum of three independent experiments. All assays were performed in triplicate to ensure reproducibility. Statistical significance ($p < 0.0001$) was assessed using one-way ANOVA followed by Dunnett's post-hoc test in GraphPad

Prism 9.0. Statistical significance was defined as a p-value ≤ 0.05. All data set prent statistically significant differences with respect to the control.
